# It’s More Than a Blood Test: Patients’ Perspectives on Noninvasive Prenatal Testing

**DOI:** 10.3390/jcm3020614

**Published:** 2014-06-19

**Authors:** Ruth M. Farrell, Mary Beth Mercer, Patricia K. Agatisa, Marissa B. Smith, Elliot Philipson

**Affiliations:** 1 Department of Bioethics, Cleveland Clinic, 9500 Euclid Avenue JJ60, Cleveland, OH 44195, USA; E-Mails: mercerm@ccf.org (M.B.M.); agatisp@ccf.org (P.K.A.); 2 Department of Obstetrics and Gynecology, Cleveland Clinic Women’s Health Institute, 9500 Euclid Avenue A81, Cleveland, OH 44195, USA; E-Mail: philipe@ccf.org; 3 Genomic Medicine Institute, Cleveland Clinic Center for Personalized Genetic Healthcare, 9500 Euclid Avenue NE50, Cleveland, OH 44195, USA; E-Mail: smithm31@ccf.org

**Keywords:** noninvasive prenatal testing, patients’ perspectives, patient decision making

## Abstract

Noninvasive prenatal testing (NIPT) offers pregnant women a new risk assessment tool for fetal aneuploidy that is superior to conventional screening tests. We conducted focus groups with women who were currently pregnant or had recently delivered in the past year to characterize their perspectives about NIPT and to explore factors they would consider during decision making about its use. Women identified accuracy, early timing, testing ease, and determination of fetal sex as advantages of NIPT over other screens, and the noninvasive method of NIPT as an advantage over diagnostic tests. False positive and false negative results, anxiety, cost and insurance coverage were seen as disadvantages of NIPT. Women who do not want fetal aneuploidy information most likely will not undergo NIPT, despite its advantages over other screening tests. However, given its advantages, the decision to have NIPT is straightforward for women who want genetic information about the fetus. Women emphasized the need to make autonomous, private, and informed choices about NIPT, as they would with any prenatal genetic testing option. These perspectives may guide clinicians to conduct effective and clinically relevant counseling with pregnant women who consider utilizing this new genetic technology.

## 1. Introduction

Noninvasive prenatal testing (NIPT) is changing paradigms about accessing and utilizing fetal genetic information to guide antepartum care [1,2,3,4]. This new prenatal genetic test provides genetic information about the fetus in early pregnancy by measuring cell-free fetal DNA in the maternal blood. Using this test, it is possible to identify the risk of Trisomy 21, 13, and 18, as well as fetal sex aneuploidy [5,6,7]. Major advantages of NIPT include a superior detection rate and lower false positive rate compared to the first trimester screen and/or the quadruple screen and the ability to be performed as early as 10 weeks gestation. Although NIPT is currently utilized as a screening test, cell-free fetal DNA technology may have diagnostic capability in the future that can be used to confirm the presence or absence of fetal aneuploidy and, eventually, to obtain a detailed fetal genetic and genomic profile. The clinical application and potential of NIPT is great. Studies show that both patients and healthcare providers are interested in this test and incorporating it into prenatal care [8,9,10]. 

Although there are clear advantages to NIPT, there are also limitations: NIPT is not a diagnostic test, and follow-up invasive testing is required to confirm the presence or absence of aneuploidy. As of yet, the positive predictive value of NIPT has not been well established in women considered low-risk by age or reproductive history or with multiple gestations. In addition, data regarding the source and chance of a false positive result in different populations has yet to be well determined [7,11]. 

It is unclear how pregnant women view the value and limitations of NIPT, both on its own and compared to other prenatal tests, in addition to factors that inform their decision-making. However, it is critical for clinicians to have this information to enable them to provide an effective and clinically relevant counseling process with pregnant women that supports a patient-centered, informed decision about NIPT, as well as other prenatal testing options. To address this issue, we developed a study to determine how pregnant women conceptualize the utility of NIPT as compared to conventional screening and diagnostic tests and identify the factors they would consider in the decision-making process about its use.

## 2. Methods

Six focus groups were conducted from 14 September 2013 to 11 January 2014 to explore women’s perspectives, including their decision-making needs and preferences, on NIPT as compared to other prenatal testing options. Eligible participants were women between the ages of 18 and 45 years who received prenatal care at the Cleveland Clinic. Women who were not in the inclusion age range, who were unable to speak or read English, or were unable to provide informed consent for research participation were excluded. 

Primary recruitment took place using *eResearch*, an information technology service of the Cleveland Clinic, which utilized ICD9 codes to extract information from the Cleveland Clinic electronic medical records (EMR) and billing systems to construct two databases: one database containing the names and contact information of patients who were currently pregnant and one containing the same information for patients who had recently been pregnant and received prenatal care since the introduction of NIPT at this institution. Randomized lists of potential participants were generated from these databases. A research staff member reviewed the last prenatal care visit or delivery note in the EMR of potential study participants to avoid contacting patients who had experienced an obstetric or neonatal complication (e.g., miscarriage or fetal/neonatal demise). Letters were mailed to remaining patients to inform them about the study. In addition, informative flyers were placed in Cleveland Clinic outpatient clinics to supplement recruitment. Those who were interested in study participation contacted the research staff by email, phone, or by returning a self-addressed response card that included their contact information. Patients who expressed an interest in participating and met eligibility criteria were invited to attend a focus group at a convenient time and location. The study was approved by the Cleveland Clinic Institutional Review Board (IRB #12-901, approved 12 September 2012).

Informed consent was obtained prior to the initiation of each focus group discussion. Participants completed a brief questionnaire to collect demographic and reproductive history information prior to the initiation of the focus group. Focus groups were attended by 8–10 participants and lasted 2 hours. With the participants’ permission, the discussions were audio recorded for data analysis purposes. 

Focus groups were led by a single moderator who has extensive experience with this methodology. The moderator began each focus group by inquiring about participants’ familiarity and experience with NIPT. An educational session about prenatal testing options, including screening, diagnostic and NIPT was then presented to inform the remainder of the discussion. A moderator guide that consisted of a series of structured, open-ended questions was used to facilitate the focus group discussions. Questions emphasized the clinical context of NIPT and decision-making about NIPT alone and in comparison with conventional screening and diagnostic testing options. The moderator guide was developed in consultation with all members of the research team and in collaboration with content experts in obstetrics, maternal-fetal medicine, genetic counseling, ethics, informed consent, and medical decision-making. A pilot focus group assessed the accuracy and precision of the moderator guide probes for the research questions, which were minimally modified for use at subsequent sessions. 

### Data Analysis

Digital recordings of the focus groups were transcribed verbatim and verified for accuracy by the research team. Data analysis was an iterative and progressive process of data immersion, coding, memoing and theme identification, which is an inductive comparative process consistent with grounded theory [12,13,14]. First, through immersion, the research team identified content domains in the transcripts to create a coding tree that was used to systematically organize the data. A companion codebook was created to serve as a reference during data coding. The data analyst coded each transcript using NVivo [15] and simultaneously composed memos to record emergent themes as well as insights and interpretations of the data. The research team held weekly data analysis meetings to monitor progress, review data coding and memoing to identify areas of consensus, identify themes, and incorporate clinical perspectives throughout the analytic process. 

## 3. Results

A total of 53 women participated in six focus groups with a mean attendance of nine women per group. Ten of the participants had NIPT during their current pregnancy. The demographic characteristics of the study sample are presented in Table 1.

Three major themes about NIPT emerged from the focus group narratives: advantages, disadvantages, and key factors in the decision-making process. 

**Table 1:** Demographic characteristics of participants.

Characteristic	*N* = 53
**Age (years)**	Mean: 31.7 Range: 21–43
	*n* (%)
Advanced Maternal Age *	
Yes	16 (30.2)
No	37 (69.8)
**NIPT Testing **	
Yes	10 (18.9)
No	43 (81.1)
**Pregnancy Status**	
**Prior 2 years**	12 (22.6)
**Current**	41 (77.4)
**Gravidity**	
G0	16 (39.0)
G+	25 (61.0)
**Trimester**	
1st	2 (4.9)
2nd	22 (53.7)
3rd	17 (41.5)
**Race/Ethnicity**	
American Indian/Alaskan Native	1 (1.9)
Asian	1 (1.9)
Native Hawaiian/Other Pacific Islander	1 (1.9)
Black or African American	17 (32.1)
Hispanic	1 (1.9)
White	29 (54.7)
Other/Multiethnic	3 (5.7)
**Education**	
High School graduate or GED	6 (11.3)
Community college	10 (18.9)
College graduate	19 (35.8)
Advanced degree	18 (34.0)

* Advanced maternal age defined as 35 years of age or older at the time of delivery.

### 3.1. Advantages of NIPT

Participants described four aspects of NIPT as advantageous: accuracy, early timing, ease of testing, and identification of fetal sex. 

#### 3.1.1. Improved Accuracy without Risk

Participants valued the novel accuracy and noninvasive aspects of NIPT as the leading advantages.

Participant 1: “It’s just a blood test”.

Participant 2: “It’s so accurate and so noninvasive” (Focus Group 4).

Participants did not express any misperceptions about the function of NIPT. They clearly recognized that NIPT is not currently considered to be a diagnostic test. They also acknowledged the possibility of obtaining a false positive or false negative result. However, in general, participants perceived that NIPT could provide superior reassurance about the health of the fetus compared to that obtained from conventional screens. One woman who had NIPT commented on how she perceived the value of NIPT in her prenatal care:

“In my mind, it was just as well as diagnostic. I know it’s not. I know there is still a risk but in my mind, it made me feel better that 99% is good enough for me. If there is a 1% chance of something happening, then it’s meant to happen but 99%, I could at least breathe a little easier”.

(Focus Group 4)

Participants preferred NIPT to invasive diagnostic tests due to the absence of risk to the fetus and the pregnant woman, as well as the fear and physical discomfort associated with the chorionic villus sampling and amniocentesis procedures. Some envisioned that if confronted with the decision to have either NIPT or an invasive test such as amniocentesis, they would choose NIPT. One participant’s remarks illustrate how her perception of reduced risk favors NIPT over invasive testing:

“The fact that it’s (NIPT) not invasive, if you had to make a quick decision and it’s just a blood test then I would think: Ok. It’s just a blood test. If I didn’t have time to think about it, it’s not anything that is going to severely harm me or my baby. Then … I guess … since it’s the safest thing, that would be like the biggest thing if you had to make a quick decision and you didn’t have time”.

(Focus Group 1)

Compared to other screens, the convenience of a blood test and streamlined access to more accurate information about the risk of fetal aneuploidy were identified by participants as key factors in the decision-making process. Participants generally viewed the high accuracy of the risk assessment provided by NIPT as a way to accelerate through previously accepted algorithms of prenatal testing and advance more directly to an answer they sought about the health of the fetus. One participant described this in comparison to the utility of conventional maternal serum screens as follows:

“To me, the tests all start the same—just the result is different. So this particular result is a little more accurate, they’re saying. So … you just kind of had gone up the ladder (of prenatal testing) but the test is still the same. You are just drawing blood”.

(Focus Group 2)

#### 3.1.2. Early Timing

In addition to accuracy, the availability of the test as early as the first trimester was considered a favorable quality of NIPT. Earlier timing was viewed as a positive aspect in two ways. Some women felt that this time frame would provide the opportunity to both make key decisions about prenatal care in early pregnancy and avoid issues that could arise if this information did not become available until the second trimester.

“I think that one of the things that appeals to me about this (NIPT) is that you can do it early enough so that there is time to make a decision if it really is just … something that a woman doesn’t feel like she can handle. There is plenty of time for her to make a decision about that. It is not something that is done late in the second trimester where it is kind of past that point”.

(Focus Group 2)

For others, earlier timing was viewed as a possible way to alleviate maternal stress and anxiety at an early stage of the pregnancy. As one participant stated:

“That is what I thought was the benefit of this—not to terminate early. But we were talking about the fear and anxiety because I had done the quad (ruple) screening and had very bad results. We ended up doing an amnio (centesis) … at 20 weeks and we had been stressing this entire time not knowing what is going on”.

(Focus Group 6)

#### 3.1.3. Ease of Testing

There was general consensus among participants that another major advantage of NIPT was the convenience and simplicity with which it could be performed. One favorable aspect is that the prenatal test involves a single blood draw during the first trimester and requires no additional appointments. As one participate described it:

“You don’t have to do an extra visit for an ultrasound, so it is definitely easier”.

(Focus Group 3)

This advantage was also associated with participants’ familiarity with having their blood drawn during pregnancy. Participants commented that, due to the number of blood tests done in pregnancy, the NIPT procedure does not present uncharted territory for them to consider as part of the testing process.

Participant 1: “We’re getting so many blood tests the entire time”.

Participant 2: “Right”.

Participant 3: “We’re used to having them … an extra vial”.

Participant 1: “Every time I go in, I walk out with blood tests”.

(Focus Group 1)

“I think for me it is just a blood test, alright. I mean you get poked and prodded so much when you are pregnant … so doing another blood test is no big deal”.

(Focus Group 2)

#### 3.1.4. Identification of Fetal Sex

There was consensus among participants that the ability to obtain information about fetal sex in the first trimester, not a widely recognized aspect of NIPT, is an advantage of the test. Of those who had undergone NIPT, some were aware of this aspect prior to testing and discussed how the ability to determine fetal sex had been a factor in their decision to utilize the test.

“That is another reason why we chose to do it because we wanted to know the sex of the baby. So I knew 5 weeks before I would have known from the ultrasound and some people (asked), ‘How do you know already?’”.

(Focus Group 1)

However, others reported that they had not known prior to testing that they could determine fetal sex and only became aware of it when they were asked if they wanted know the sex when given NIPT results. These participants perceived learning this information as a “bonus” of NIPT. Participants also discussed whether women might have NIPT primarily to learn the sex of the fetus early in the pregnancy.

Participant 1: “You wonder if people are going to want the test just to find out the sex earlier”.

Participant 2: “I think it probably has something to do with it”.

Participant 3: “Oh yeah”.

(Focus Group 4).

One participant cautioned that women should be fully informed about the purpose of NIPT prior to testing. Although it might be acceptable to have the test done primarily for fetal sex identification, they advocated that women undergoing the test should be prepared for whatever else they may learn about the fetus as part of the process.

“If it (NIPT) does become more common and women are doing it just to get some of the information like the sex of the baby early on… Be careful… They give you the results over the phone and if you’re expecting to get a phone call saying it’s a boy or a girl and (they report) your baby has a 99% chance of (an abnormality)…, you need to know to expect the information. I think people need to be informed of the, ‘This is what we’re really looking for’ and that you are going to be given results on it and how they record that”.

(Focus Group 4)

### 3.2. Disadvantages

Although participants recognized the advantages of NIPT, they also felt there were disadvantages and limitations inherent to this test. 

#### 3.2.1. Risk of False Positive and False Negative Results

Although the accuracy of NIPT was viewed as an advantage when compared to other screening tests, participants acknowledged that one disadvantage of the test is that it does not provide definitive information regarding fetal aneuploidy. As part of these discussions, participants recognized that the chance of a false positive and false negative result is greatly decreased with NIPT. However, they still considered the possibility of such results as an important disadvantage and factor in their decisions about its use. There was consensus among participants that the stakes are high: important decisions are made based on the result so it needs to be “true”. The following quotes summarize participants’ perspectives:

“I think it is a scary thought to make a decision based on something that isn’t true… I would be scared to make the wrong decision or then you are worried the whole time and then your doctor was wrong. Then you have a kid and there was nothing wrong with it and you are stressing and you are worried about it and you are trying to be prepared and you don’t know what to do and there is nothing wrong with the kid so you did all of that for nothing”.

(Focus Group 3)

“I would take every test as long as I knew it was going to tell the truth. The reason that I didn’t do it with my other children was because I felt like the risk of having a problem wasn’t as high as the risk of having a false positive, in my mind. It may not be true, but in my mind, I didn’t want to freak out the whole rest of my pregnancy and then to … give birth and everything was fine and I spent the last nine months putting all of this stress on the baby and myself”.

(Focus Group 4)

#### 3.2.2. Stress and Anxiety Associated with the Testing Process

Participants spoke about the desire to embrace and enjoy the experience of pregnancy. Yet, many women characterized the prenatal testing process as stressful and fraught with feelings of uncertainty and anxiety. The increased accuracy and decreased false positive rates of NIPT did not seem to minimize these feelings. Regardless of their choice of prenatal test, women experienced anxiety when thinking about a myriad of possibilities associated with the testing process: (1) having to undergo confirmatory diagnostic testing if the NIPT result were positive; (2) preparing for a child with a genetic condition; or (3) considering the decision to terminate a pregnancy. As described by a participant:

“Because there is nothing that I can do about it and I don’t want to be stressing out the whole time and then what if they are wrong? So then I am stressing out the whole time and it is wrong… I would rather deal with it when it comes up rather than being worried about something that is out of my control. I know that I am doing everything I can right now to be as healthy as possible”.

(Focus Group 6)

#### 3.2.3. Cost and Insurance

Participants spoke about the cost of NIPT and the issue of insurance coverage as being potentially problematic for some women wanting this test. Participants thought that women may have difficulty with insurance coverage because the test is so new or because they were not eligible to have the test based on ACOG recommendations, *i.e.*, maternal age of 35 years or greater at the time of delivery. Those who had undergone testing shared their experiences with insurance coverage and billing. One participant, who works in health care, shared that she knew of patients who cancelled their NIPT because of a lack of insurance coverage:

“There are a lot of patients that schedule it and say, ‘I’m going to call my insurance company afterwards’. And they call back and cancel because their insurance company was like, ‘There’s no way we are paying for this’. So, they’re not willing to take that leap but you know, everybody is hoping within X amount of time, they’ll be like this is just part of normal prenatal care”.

(Focus Group 4)

### 3.3. Decision Making about NIPT

Focus group participants also discussed a number of factors that could impact women’s decision-making about this test. 

#### 3.3.1. Autonomy and Privacy

The issues of women’s autonomy and privacy in the decision-making process about NIPT emerged during an early focus group discussion and were further explored in subsequent groups. There was agreement among participants across focus groups that final decisions regarding prenatal genetic testing reside with the pregnant woman. Many participants disclosed that in their experiences with prenatal testing, their partners agreed with them regarding their decision about testing. When there was disagreement, participants felt that they should have the final decision to test or not. Furthermore, they suggested that women could undergo NIPT if they desired the information without telling their partners. One exchange among participants discussed such as scenario:

Participant 1: “You could get the results and not tell anyone”.

Participant 2: “Right”.

Participant 3: “Do it behind their (partner’s) back”.

Participant 4: “That is exactly what I would do, at least if I felt that I needed the results. I would get them and not mention it”.

Participant 5: “Not ‘til that bill comes” (Focus Group 1).

The issue of privacy was also discussed. Participants emphasized that pregnant women should be able to control the disclosure of information throughout their pregnancy. This participant commented on privacy in the context of NIPT:

“With it being a sensitive subject people may not be willing to discuss it, especially if it (NIPT) comes back positive. That may not be something they are willing to discuss with their relatives. So having that privacy is definitely an advantage”.

(Focus Group 3)

In contrast, another participant spoke about the challenges of maintaining privacy when additional procedures are a part of the prenatal testing process:

“But in my husband’s case, his mom is, I am going to call her a concerned mother but to me she is just nosey, she would be the one asking ‘What is going on? What are we having done?’ Even when we just had the little ultrasound done to … test for Down syndrome. That time she was asking questions about it and she wanted me to have the amnio(centesis) done to check… We didn’t even say anything to her about it. She wants to know. So in those cases, what do you say? I can’t tell you? Do you lie”?

(Focus Group 2)

#### 3.3.2. The Need to Consider Test Implications Remains Unchanged

Despite the perceived advantages of NIPT, consideration of the consequences of prenatal genetic testing was not a diminished factor in the decision-making process. Participants articulated the need to consider the implications of obtaining fetal genetic information prior to undergoing testing. While they recognized that NIPT offers distinct advantages over other screens, pretest knowledge of their choices in light of test results would be an important consideration for participants. As illustrated by the following participant:

“I would need to know the purpose of it (NIPT) too... Because for me, I would not terminate so I wanted to know, why do you need to know this if I’m not going to terminate the pregnancy? He (the doctor) explained to me that it was good to know because there are more risks during delivery and things that they need to know about. So that was, for me, … a tipping point to even why because I didn’t have anything done with my other three (children). I didn’t have any testing at all. So, I need to know the purpose. What would I do with the results?”.

(Focus Group 4)

Because of the importance of this consideration, participants recommended that clinicians clearly communicate to patients the options available if they received a negative or positive result. One participant suggested that, as part of the counseling process for NIPT, clinicians should ask women to imagine either result and what they would do:

“I think it’s useful to go through the scenario … if it’s positive *versus* if it’s negative. What would you do in each situation or what would you be advised to do in each situation—to walk through those (options). That way, when the phone call comes with the results, it’s not necessarily … what do I do now?”.

(Focus Group 4)

#### 3.3.3. Tipping the Scales

In general, focus group participants indicated that the availability of NIPT would not tip the scales towards testing if a woman does not desire to obtain this type of information. The general consensus was that if women do not want fetal aneuploidy information, they would probably not have NIPT, despite its perceived advantages over other screening tests.

“I feel like a lot of women who decide they don’t want any testing done—it’s usually because it wouldn’t change the outcome whether they knew something was wrong or not. So I think offering this would be the same kind of thing… If they are not going to do anything different then if it is a blood draw or if it is a scan, I still wouldn’t do it. I don’t know how it would change their mind if that was their reason for not doing a screening. If you don’t want to do an amino(centesis) because it is invasive, then yeah, this is a lot simpler and easier”.

(Focus Group 6)

However, participants expressed that if women are interested in having prenatal genetic testing, the decision about NIPT is an easy one to make. One woman’s reflection on her experiences with NIPT and other prenatal genetic tests illustrates this viewpoint.

“I had the test as well—the NIPT test. Three years ago when I had my son, the blood test (NIPT) wasn’t available so I had a quad(ruple) screen that came back with markers for Down syndrome so I went through and actually did the amniocentesis for him… When they said this was available, it was a no-brainer. You’re going to do a blood test for me and I get the same type of information… It was just an easy choice for me to make, to get the same information with a lot less risk”.

(Focus Group 1)

#### 3.3.4. Preserving the Opportunity to Make Deliberate and Informed Choices

The need to make deliberate, informed choices emerged during the discussions. One aspect of this pertained to the ability to make choices about testing that reflect the needs and preferences of the pregnant women. One participant shared her experience with NIPT in terms of knowing that testing was a choice but feeling as though it was expected of her:

“I don’t think it was explained to me that way or that specifically. I almost feel like my experience was a little more like <name>’s, where they said, ‘Ok, these are the tests available. Let’s get these ordered and you can call and sign up’. And it is kind of like you are almost expected to do it”.

Moderator: “Did you feel like you had a choice?”

“I feel like I knew I had a choice. I would have chosen it anyway. I was fine to have any testing done because it was more about being prepared… and to know more about it. It was more like, ‘Ok. Here are a couple tests. Here is what they test for… They are ordered. You can call and schedule them now’”.

(Focus Group 3)

Other participants shared their thoughts that women may be perceived as noncompliant when they do not undergo recommended prenatal tests, a standard part of care. As expressed by one participant:

“Offered but then again, when you don’t, it seems like if you don’t want to do it (prenatal screening), you are not compliant”.

(Focus Group 3)

Because NIPT requires a single blood draw, some participants recognized the possibility that women could have NIPT without full awareness that the test is being performed. Participants suggested this could occur because pregnant patients are accustomed to the practices and patterns of prenatal care. For some, this possibility was linked to the routine nature of undergoing blood tests throughout pregnancy. Those who shared this perspective could imagine NIPT being one among many blood tests ordered:

Moderator: “Is it possible that a woman could have NIPT and not even know she’s even having the test?”

Participant 1: “Sure. They take nine vials of blood at your first check-up”.

Participant 2: “That’s right”.

Participant 3: “We don’t know everything they are testing for”.

(Focus Group 4).

Participants also thought being uninformed about testing would be unethical due to lack of disclosure, lack of consent, and privacy violation. When probed about reactions to the possibility of having NIPT without their knowledge, participants responded as:

Participant 1: “That would be bad”.

Participant 2: “Not good”.

Participant 3: “That is not ethical, for sure”.

(Focus Group 1).

Others thought that the possibility that one could have NIPT without full awareness could be an unintended result of how women receive and process information in the context of their prenatal care. Participants spoke about feeling overwhelmed with information:

“I took the test (NIPT) and I knew I was going to … once I found out about it. But, I guess I didn’t really process or didn’t absorb if they (the results) came back negative or there are markers… a result saying there is some likelihood (of aneuploidy) and what my next step was. It may have been in the literature or the doctor might have talked about it but it certainly wasn’t something that I had really considered...”.

(Focus Group 3)

However, participants agreed that, although the clinician is responsible for presenting patients with all of their options so they make fully informed decisions, women have the responsibility to ask questions, understand their options, and make informed decisions. As expressed by one participant:

“To be fair to my healthcare provider, they have always told me (about testing options). I haven’t really absorbed everything, you know? I have had pregnancy brain since the beginning. But, I read my consent forms. I do that and if I am not going to read a consent form, I am not going to read any other pamphlet that you hand me. But when I have a consent form to sign and it says, for example, you are taking, or you agree to the NIPT, I have a chance to stop and say, ‘Answer this question for me’ or, ‘Don’t do the test’. That option is there. Yes. I feel rushed, but I still have that option”.

(Focus Group 1)

## 4. Discussion

As NIPT is quickly becoming available for clinical use, the need for post market data regarding women’s opinions of this new test is paramount. The objective of this study was to explore women’s perceptions of the utility of NIPT in comparison to conventional screens and diagnostic tests as well as other factors they would consider during decision-making. To our knowledge, this is the first report of women’s perceptions about NIPT, including the perceptions of those who were early adopters. Our study demonstrates that the unique and appealing aspects of NIPT do not simplify women’s decision-making process about its use or reduce the support women need to make informed choices regarding which test would best match their needs and preferences. 

A major finding of our study is that the noninvasive aspects of NIPT would not influence women who are otherwise not inclined to undergo prenatal genetic testing. However, for those who were interested in prenatal genetic testing, the ease with which NIPT could be performed was valued. Because blood tests are a routine part of prenatal care, participants were concerned that women may undergo NIPT without making a deliberate, informed choice about it and the ethical implications of such a situation. There was also a sense that women may feel expected to undergo testing due to the ease and convenience of NIPT. These findings emphasize the need for effective educational and consent mechanisms to ensure women make informed, value-reflective choices about this new testing option. These efforts should include a description of test sensitivities, not only for Trisomy 21, but also for Trisomy 13 and 18, to help patients understand these concepts [1,2,3,4] in addition to emerging data about the rate and etiologies of false positive results [16]. The importance of pretest counseling and informed consent to pregnant women has been noted by other authors [17,18,19,20,21]. However, a survey of obstetric care providers showed that they were less likely to believe that informed consent should be obtained prior to NIPT testing [22]. Although informed consent discussions pertaining to prenatal genetic testing most often come into play when the risks of invasive testing are discussed [23,24,25], these issues highlight the need to develop formal informed consent processes for noninvasive testing.

A surprising finding was that participants voiced concern about the possibility and consequences of a false positive result, despite acknowledging that the chance of this occurring is significantly decreased compared to other screening tests. For participants, this continued to be an important factor in their decision-making about NIPT. Women in this study were not naive to the concept of a false positive result, an awareness that could have been developed from their own counseling experiences from a prior pregnancy or from a general dialogue among family, healthcare providers, and peers regarding conventional screening options. Concerns about the chance and significance of a false positive result could be a carryover of attitudes and beliefs concerning conventional screens [26]. It may also be due to how women conceptualize risk, a notoriously difficult concept to effectively convey in provider-patient discussions. Although the 1% chance of obtaining a false positive result from NIPT is less likely than the typical 5% chance associated with conventional screens, women may have difficulty contextualizing this numerical value into personal deliberations about prenatal testing [27]. While further studies will be needed to elucidate this finding, other studies clearly demonstrate the impact of concerns and risks perceptions associated with prenatal genetic testing on women’s experiences and decisions during pregnancy [28,29,30]. 

Our study also demonstrates that, despite the ease and accuracy of NIPT, women continued to characterize the testing experience as stressful, filled with uncertainty, and anxiety provoking. Although the improved accuracy of NIPT appeared to reduce these concerns, uncertainty regarding the health of the fetus and associated anxieties persist independent of how fetal genetic information is obtained. These concerns did not arise as much from risks or doubts about the performance of NIPT, but instead when women examined their own values and beliefs concerning pregnancy termination and raising a child with a serious medical condition in the context of prenatal testing. Thus, these concerns would most likely exist regardless of how the fetal genetic information is obtained.

The capability and availability of NIPT also raised some new concerns that have not been previously described. One issue pertained to privacy. Participants underscored the need for pregnant women to make autonomous and private decisions regarding prenatal genetic testing, particularly when there was disagreement with their partners. Because prenatal genetic testing touches upon polarizing issues of disability and termination, the ability for women to make intimate decisions about the pregnancy is paramount [31,32]. The ease and convenience of NIPT may enhance women’s decision-making autonomy and privacy with regards to prenatal genetic testing. Specifically, the procedural single blood draw simplifies the testing process so that woman may have testing without the knowledge of others, including partner and family. Thus, NIPT may present women with the option for a new level of privacy that is not as easily obtained by other forms of testing.

The ability to determine fetal sex early in the pregnancy without the need for invasive testing was perceived as “a bonus” for women. However, participants also raised caution about women’s motivations for undergoing NIPT and the decisional implications of receiving information for which they had not been prepared. This finding also underscores the need for prenatal education that provides comprehensive information about the purposes of the test and counseling so that women understand the implications of the results.

Our study is the first to bring to light women’s opinions about NIPT and its incorporation into prenatal care. However, we acknowledge that the study has some limitations, as these qualitative data provide insight into only one community of women’s acceptance of NIPT and their informational and decision-making processes necessary for informed uptake of this new technology. Nonetheless, our sample population included both women who had personal experience with NIPT as well as women who were introduced to this new testing modality during the focus group. Although our sample size was limited, our findings from a group of women with diverse reproductive histories and ethnicities provide insight into the complexity of the decision-making process women may confront when considering the use of emerging prenatal genetic technologies. 

## 5. Conclusions

How pregnant women conceptualize the utility of NIPT, compare it with other prenatal genetic testing options, and make decisions about its use provides valuable information to clinicians. Participants in our study valued the accuracy, early timing, ease of testing, and determination of fetal sex as key advantages of NIPT over other screens and the noninvasive nature of NIPT over diagnostic tests. Despite these advantages, participants agreed that women who do not want fetal aneuploidy information would likely not have NIPT. The possibility of a false positive result would continue to be an important factor in decision making about NIPT even though the test is far more accurate than alternative screening choices. Finally, participants underscored the need for women to make deliberate, informed choices about NIPT, as they would with any prenatal genetic testing option. Thus, evidence-based and clinically relevant mechanisms must be in place to ensure pregnant women have the support they need when considering this novel testing option. 

## Appendix

**Moderator Guide**

**Familiarity with NIPT**

Have any of you heard of NIPT? What do you know about it? Where did you learn about it? What were your initial reactions? (If participant(s) volunteer that they had NIPT, ask them if they are comfortable sharing their experiences with the test.)

**Presentation about NIPT and Other Prenatal Genetic Testing Options**

Overview of Down syndrome, trisomy 18 and trisomy 13.
Overview of prenatal genetic testing options.
Overview of NIPT and comparisons to conventional screens and diagnostic tests.
Questions and Answers.

**Reactions to NIPT**

What are your thoughts about NIPT?
What are the advantages of NIPT? What are the disadvantages of NIPT?
How does NIPT compare to other prenatal tests for chromosomal abnormalities? How is it similar? How is it different?

**Decision Making and Informed Consent**

What information would you need to decide if NIPT is right for you?
How does the accuracy of NIPT affect decisions about whether or not to get the test?
Does the fact that it’s a blood test affect how you would weigh the benefits and risks of the test?
How can doctors and midwives help women make informed decisions about this test as it changes over time?
